# Visualization and quantification of inhomogeneous and anisotropic magnetic fields by polarized neutron grating interferometry

**DOI:** 10.1038/s41467-019-11590-2

**Published:** 2019-08-22

**Authors:** Jacopo Valsecchi, Ralph P. Harti, Marc Raventós, Muriel D. Siegwart, Manuel Morgano, Pierre Boillat, Markus Strobl, Patrick Hautle, Lothar Holitzner, Uwe Filges, Wolfgang Treimer, Florian M. Piegsa, Christian Grünzweig

**Affiliations:** 1 Laboratory for Neutron Scattering and Imaging, Paul Scherrer Institut, Villigen, Switzerland; 2 University of Geneva, Geneva, Switzerland; 3 Electrochemistry Laboratory, Paul Scherrer Institut, Villigen, Switzerland; 4 Niels Bohr Institute, University of Copenhagen, Copenhagen, Denmark; 5 Laboratory for Scientific Developments and Novel Materials, Paul Scherrer Institut, Villigen, Switzerland; 6 Beuth Hochschule für Technik, University of Applied Sciences, Berlin, Germany; 7 Laboratory for High Energy Physics, Albert Einstein Center for Fundamental Physics, University of Bern, Bern, Switzerland

**Keywords:** Magnetic properties and materials, Imaging techniques

## Abstract

The intrinsic magnetic moment of a neutron, combined with its charge neutrality, is a unique property which allows the investigation of magnetic phenomena in matter. Here we present how the utilization of a cold polarized neutron beam in neutron grating interferometry enables the visualization and characterization of magnetic properties on a microscopic scale in macroscopic samples. The measured signal originates from the phase shift induced by the magnetic potential. Our method enables the detection of previously inaccessible magnetic field gradients, in the order of T cm^−1^, extending the probed range by an order of magnitude. We visualize and quantify the phase shift induced by a well-defined square shaped uniaxial magnetic field and validate our experimental findings with theoretical calculations based on Hall probe measurements of the magnetic field distribution. This allows us to further extend our studies to investigations of inhomogeneous and anisotropic magnetic field distribution.

## Abstract (web summary)

The magnetic field imaging on microscopic scale is of great importance to fundamental research as well industrial applications. Here the authors show the capability to visualize and characterize the magnetic properties with 100-micrometer resolution in macroscopic samples using a cold polarized neutron beam in neutron grating interferometry.

## Introduction

Neutrons have played an essential role in studying and probing magnetic phenomena in matter due to their intrinsic magnetic moment and charge neutrality^1,2^. The information retrieved from neutron experiments can be distinguished into two large classes. On the one hand, the reciprocal space data from neutron scattering methods covers the Angstrom and nanoscale, ranging from 0.1 to 500 nm without direct spatial resolution capabilities^3–6^. On the other hand, real-space data, such as the one retrieved by neutron imaging measurements, can resolve macroscopic structures. Utilizing polarized neutrons, neutron imaging has proven to be a powerful tool for retrieving spatially resolved information about magnetic field distribution^7,8^. Imaging with polarized neutrons has progressed in recent years and different methods have been developed, capable of analyzing and directly visualizing local magnetic interactions^9^. The technically simplest realization of neutron imaging with a polarized beam is called neutron depolarization imaging (NDI)^7,8,10^. It enables to investigate whether the magnetic sample contains non-parallel field components with respect to the initial beam polarization, which cause depolarization effects in the neutron beam. This method has proven to be a powerful tool in many different applications such as mapping of the Curie temperature of samples undergoing a ferromagnetic–paramagnetic phase transition^11,12^. A somewhat more sophisticated approach is applied for the polarized neutron imaging, where the beam can be assumed to remain polarized even after the interaction with the sample and the local spin rotation is analyzed. This spin state analysis technique relies on the physical phenomenon called Larmor precession, in which the polarization vector of the neutron beam precesess around the field vector and enables keeping track of the precession angle. Thus, one can retrieve direct information on the magnetic field distribution, which was demonstrated by the direct visualization of the field produced by an electrical coil at different current values and subsequently applied to observe magnetic fields trapped and flux pinning behavior in the bulk of superconducting samples^13–19^. Neutron spin phase imaging, based on the Ramsey principle, which keeps track of any additional precession angles due to a sample shifts, proved the feasibility of quantitative imaging of magnetic fields^20^. The latest development in the field of polarization imaging is the so-called polarimetric neutron imaging^8,21^. This technique is capable of full 3D tomographic reconstructions of magnetic vector fields. This is achieved by spatially resolved measurements of the 3D rotation state of the polarization vector for all three orthogonal incoming polarization directions and is suited to take advantage of time-of-flight capabilities at advanced pulsed sources^8,9,22–24^.

A common feature of imaging techniques with polarized neutrons described so far is that these are well suited for weak magnetic fields, up to few mT, characterized by smooth gradients distribution up to maximum ≈0.1 T cm^−1^^8^. These limits are due to the intrinsic resolution of these techniques in solving the integrated Larmor precession angle, which is the physical quantity carrying the information about the magnetic field distribution. A first attempt to measure stronger magnetic fields based on differential phase effects has been performed with a double crystal diffractometer in a scanning mode approach. This technique relies on the detection of the neutron wave packet’s phase shift induced by the magnetic field. Since it is a slit scanning imaging method its major drawbacks are the strongly limited field of view, the resolution, and the long exposure times even for single slices^25^.

Here we introduce an imaging-based approach through polarized neutron grating interferometry (pnGI), which utilizes polarized neutrons in nGI, thus extending the covered range of magnetic field gradients of former techniques by an order of magnitude. In general nGI provides simultaneously three different contrast signals: the conventional transmission image (TI), the differential phase contrast image (DPCI), and the dark-field image (DFI). The first nGI experiment was conducted to visualize and quantify the neutron wave packet’s phase shift induced by nuclear interaction from the DPCI^26^. Recently, most of the studies with nGI have mainly focused on the small-angle scattering information yielded by the DFI^27^. The neutron DFI signal was then adopted for tomographic investigations providing non-magnetic, spatially resolved information about the distribution of micrometer and sub-micrometer-sized structural formations^28^. Another main field of research by means of DFI is the visualization of magnetic domain structures in bulk ferromagnetic grain-oriented electrical steels^10,29–35^. An emerging topic of interest for DFI is the investigation of local magnetic phenomena in superconductors, such as the domain distribution in the intermediate state of Lead^36^ and the morphology of lattice domains in type-II superconductor^37,38^. Significant progress has been made in DFI, especially providing full structural information of microstructures through the so-called sub-pixel correlation length imaging (*ξ*DFI)^39–42^.

In this paper, we demonstrate how the introduction of polarized neutrons to nGI can extend applications and be efficiently used to retrieve quantitative information about the quantum-mechanical phase shifts of neutron de Broglie wave packets induced by magnetic interaction with the sample. This will enable us to visualize and quantify the spatial distribution of strong magnetic fields and field gradients.

Considering a magnetic vector field, the scalar potential *V*_mag_(**r**) can be expressed as^2^:

1 $$V_{\mathrm{mag}}({\mathbf{r}}) = - \widehat {\boldsymbol{\mu }}_{\mathrm{n}} \cdot {\mathbf{B}}({\mathbf{r}}),$$

where $\widehat {\boldsymbol{\mu }}_{\mathrm{n}}$ is the neutron magnetic moment operator and **B**(**r**) is the magnetic field.

Therefore, depending on the orientation of the spin state of the neutron with respect to the magnetic field, the neutron gains or losses kinetic energy according to Δ*E* = ±*μ*_n_*B*; hence, the induced phase shift of the wavefront is equal to^43^:

2 $${\mathrm{\Delta \Phi }} = \pm \frac{{\mu _{\mathrm{n}}Bm_{\mathrm{n}}\lambda D}}{{2\pi \hbar ^2}},$$

where the ± is due to the Zeeman splitting^2^ of the eigenstates of the Pauli spin operator when they are aligned parallel and anti-parallel to the external field. Where *μ*_n_ = −9.6623647 × 10^−27^ J T^−1^, *m*_n_ is the neutron mass, *λ* the wavelength, *D* the path length through the magnetic field, and ℏ = 1.0545718 × 10^−34^ J·s the reduced Plank constant, respectively.

In order to fully understand the general case of the dot product in Eq. (1) one has to consider the interaction of a neutron spin along its trajectory in an inhomogeneous magnetic field. Depending on the smoothness of the gradient and the neutron energy, two different scenarios can occur: either an adiabatic or a non-adiabatic transition.

The adiabatic regime is defined by the condition^2^:

3 $$\frac{{\frac{{{\mathrm{d}}({\mathbf{B}}/|{\mathbf{B}}|)}}{{{\mathrm{d}}t}}}}{B} = \frac{{\frac{{\mathrm{d}({\mathbf{B}}/|{\mathbf{B}}|)}}{{{\mathrm{d}}x}}\frac{{{\mathrm{d}}x}}{{{\mathrm{d}}t}}}}{B} < < \gamma B = \omega _{\mathrm{L}},$$

where $\frac{{{\mathrm{d}}({\mathbf{B}}/|{\mathbf{B}}|)}}{{{\mathrm{d}}t}}$ is the frequency with which the direction of **B** changes. For a static magnetic field this depends on the gradient of the magnetic field **B** and $\frac{{{\mathrm{d}}x}}{{{\mathrm{d}}t}}$ the velocity of the neutron as it concerns the field as experienced by the neutron*. ω*_L_ is the Larmor frequency and *γ* the gyromagnetic ratio of the neutron. According to Eq. (3) if the direction and the magnitude of the magnetic field gradually change in a sufficiently small and in a continuous manner, the adiabatic condition is fulfilled. This means when the system evolves close to the equilibrium the neutron spin is coupled to the magnetic field and the parallel polarization component is conserved. Instead in the non-adiabatic regime the neutrons spin oscillates between the two possible spin states with respect to the applied field, resulting in the manifestation of a precession of the spin vector around the magnetic field vector known as Larmor precession and with the Larmor frequency. This non-adiabatic transition of the spin vector is interpretated as an interference effect of the quantum superposition of the two possible spin states up |↑〉 and down |↓〉.

Here we demonstrate the capabilities of pnGI for visualizing and quantifying inhomogeneous and anisotropic magnetic fields based on differential phase contrast and adiabatic spin transition, which applies to the coupling between guide field and sample. In a initial experiment, we investigated a well-defined square-shaped uniaxial magnetic field-oriented parallel to the neutron spin polarization of the incident beam. The field was produced by strong neodymium permanent magnets. The study extends to investigate an inhomogeneous and anisotropic magnetic field distribution in various orientations with respect to the incident polarization direction.

## Results

### Grating interferometry with polarized neutrons

**Fig. 1:**
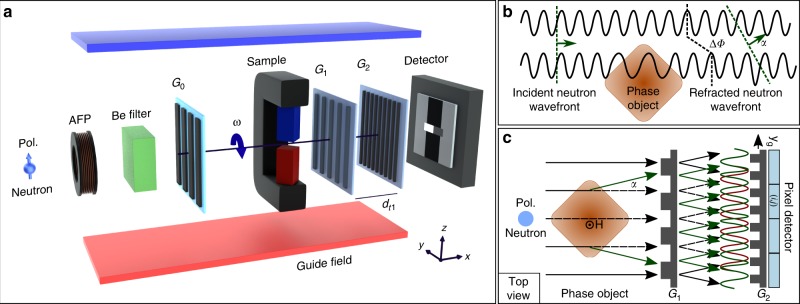
Experimental setup and principle of polarized neutron grating interferometry (pnGI). **a** Sketch of the pnGI setup for polarized neutrons. The setup consists of an adiabatic fast passage spin flipper (AFP), a beryllium filter (Be filter), a source grating *G*_0_, a phase grating G_1_, an analyzer grating *G*_2_, a detector, and a guide field system. The magnetic sample is placed between *G*_0_ and G_1_, the North and the South poles are depicted in red and blue, respectively. The parameter *ω* is the rotation angle of the gratings and the sample around the *x*-axis, the configuration in the sketch corresponds to *ω* = 0°, and *d*_*t*1_ is the Talbot distance. **b** Phase shift, Δ*Φ*, of the neutron wavefront induced by the magnetic interaction with a phase object, refracted by an angle *α*. **c** Schematic top view of a pnGI setup depicting the perturbations of the incident neutron wavefront, induced by refraction on a phase object in the beam, which lead to local displacement of the interference pattern. The intensity modulation is detected by each (*i*, *j*)-pixel on the matrix detector. The phase-stepping approach is adopted by scanning one grating along the transverse direction, *y*_g_. Further information about phase-stepping method for pnGI and contrast mechanism can be found in the Supplementary Note 1

The pnGI experiments have been carried out at the polarized cold neutron beamline BOA (Beamline for neutron Optics and other Applications), at the Swiss Spallation Neutron Source (SINQ), and at the Paul Scherrer Institut (PSI)^44^. A schematic of the pnGI setup is shown in Fig. 1a–c. The neutron interferometer is composed by an absorbing source grating (*G*_0_), the phase grating (*G*_1_), and the analyzer grating (*G*_2_)^45^. The sample is placed between *G*_0_ and *G*_1_, as close as possible to the detector in order to reduce the geometrical image blurring. The neutron spin direction can be set to be parallel or anti-parallel with respect to the initial polarization by an adiabatic fast passage spin flipper (AFP)^44,46^. A beryllium filter (Be filter) is used as a cold neutron filter. We adopted the phase-stepping approach for images acquisition of the pnGI Fig. 1c. Both spin states are reconstructed with the software TaPy based on Marathe et al. algorithm^47,48^. The data acquisition of both spin states is necessary to characterize the polarization of the neutron beam and, moreover, to discriminate between the phase shift induced by the nuclear interaction, which is not spin dependent, and the magnetic one, which is spin dependent. For more detailed explanation we refer to the Supplementary Note 1.

### Phase shift induced by a homogeneous uniaxial magnetic field

**Fig. 2:**
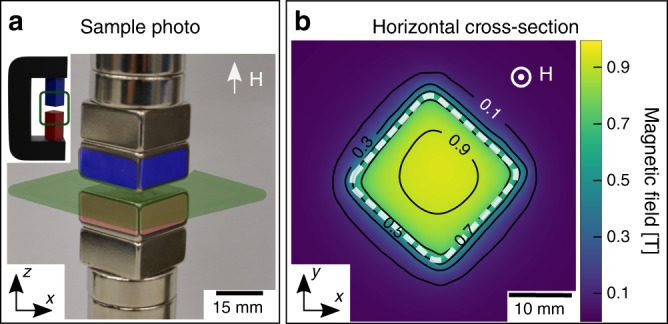
Homogeneous, well-defined, square-shaped, and uniaxial magnetic field characterization. **a** The inset shows the sketch of the permanent magnets and the iron yoke along the beam direction. The photograph displays the air gap (4 mm) between the square-shaped pole shoes (blue and red), which produce a square-shaped uniaxial magnetic field and the 45° orientation permanent magnets around the vertical axis. **b** Hall probe map of the measured magnetic field between the squared-shaped pole shoes, as depicted by the green horizontal cross-section plane in **a**. The white dashed line indicates the edges of the permanent magnets

Through pnGI we visualize and quantify the neutron wave packet’s phase shift induced by a homogeneous, well-defined, square-shaped, and uniaxial vertical magnetic field. Figure 2a shows a sketch and a photograph of the sample. The magnetic field in the probed air gap between the two pole shoes is produced by two square-shaped neodymium permanent magnets (NdFeB) with an edge length of 25 mm as pole shoes. In order to obtain a flux closure in the magnetic circuit and to avoid beam depolarizing stray fields, the permanent magnets are housed in an iron yoke, as shown in the inset of Fig. 2a. The magnetic field produced in the air gap can, to a first approximation, be considered being **B** = (0, 0, *B*_*z*_). The Hall probe measurement presented in Fig. 2b shows the morphology of the magnetic field distribution, highlighting its well-defined square shape. The strength of the magnetic field within the square shape is characterized by a quasi-homogeneous distribution, ranging from 0.9 T in the center to 0.75 T near the edges, while outside the pole shoes it drops sharply without discontinuity. Nevertheless, as we will show, the neutron spin aligns to the sample field direction fulfilling the adiabatic condition of Eq. (3).

**Fig. 3:**
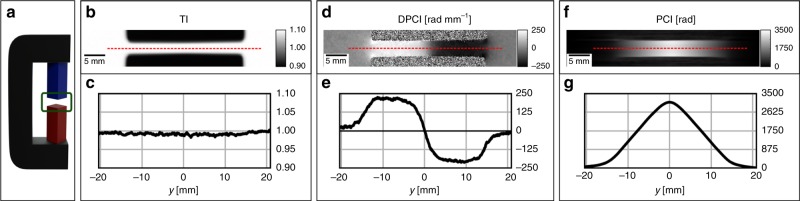
Transmission image (TI), differential phase contrast image (DPCI), and phase contrast image (PCI) results of a homogeneous and uniaxial magnetic field. **a** Sketch of the yoke sample and the green box highlights of the region of interest. **b** Conventional attenuation image, the two black regions are the neodymium permanent magnets of the pole shoes. **c** Horizontal line profile of the TI. **d** DPCI, the strong contrast emerges in between the two pole shoes and sharply drops outside the air gap. **e** DPCI line profile showing the two-step opposite values due to the constant gradient of the magnetic field distribution. **f** Integrated PCI. **g** PCI line profile showing the triangular shape characteristic of a constant gradient. The images refers to a single spin state orientation

An experiment has been performed with the following arrangement of the pnGI setup and sample: the grating lines, the magnetic field produced by the yoke and guide field are all oriented along the *z*-axis and parallel to the incoming neutron polarization. We refer to this configuration as *ω* = 0°, as depicted in Fig. 1. In this case Eq. (1) turns into the direct product of the lengths of two vectors. The sides of the square-shaped magnetic field cross-section as depicted in Fig. 2b are oriented 45° to the incident beam along the *x*-axis as illustrated in Fig. 3a. This leads to a constant gradient in the path ${\int} B(s){\mathrm{d}}s$ across the probed field, however, with opposite sign left and right from the mid position. The field integral is proportional to the induced magnetic phase shift of the neutron wave function corresponding to Eq. (2) and the gradient implies refraction through the corresponding distortion of the incident plane wavefront.

Figure 3 depicts an illustration of the region of interest and the results of this measurements in three images and corresponding cross-sections for: TI in Fig. 3b, c, DPCI in Fig. 3d, e, and PCI in Fig. 3f, g. The TI in Fig. 3b, c, the attenuation image naturally shows no response in the air gap of the field region, but only depicts the outline of the pole shoes which fully attenuate the beam.

The DPCI, Fig. 3d, displays a strong contrast in between the pole shoes, which arises from the constant gradient of the induced phase shift, but of opposite signs for both sides of the magnetic field. Accordingly, it appears dark on one side and bright on the other.

The line profile in Fig. 3e highlights the two constant values, ±240 rad mm^−1^. The transversally integrated DPCI provides the PCI, Fig. 3f, showing the constant gradient of the phase shift induced at each side of the diagonal square magnetic field region and smooth behavior at the edges. The latter is best displayed in Fig. 3g, illustrating the decay of the field close to the corners.

The relation between interferometric phase shift *θ* of the detected pixelwise intensity oscillations and the induced magnetic phase Φ in the neutron wavefront phase profile, see Supplementary Note 1, can be expressed as^26^:

4 $$\theta = \frac{{\lambda d_t}}{{p_2}}\frac{{\partial \Phi }}{{\partial y}}$$

for the DPCI results, that is, the interferometric phase shifts *θ* measured, and correspondingly Eq. (2) for the phase (PCI) the measured values corresponding to a *B* value of 1.3 T. However, since the induced magnetic phase Φ, Eq. (2), the interferometric phase *θ*, Eq. (4), and also the visibility of the interferometer are wavelength-dependent^48^, the effect of utilizing a broad spectrum has to be taken into account, both for the evaluation process of the pnGI data and for the simulation-based calculations for validation of the results.

**Fig. 4:**
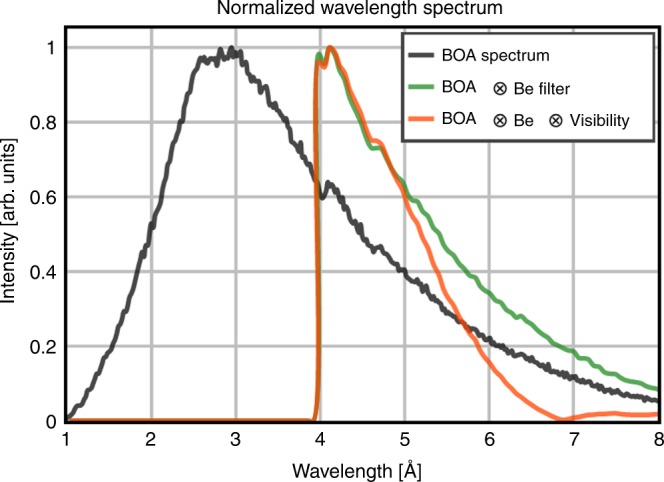
Wavelength spectrum. Normalized wavelength spectrum of the BOA (Beamline for neutron Optics and other Applications) beamline (black), with the beryllium filter (green) and its convolution with the visibility function calculated for the polarized neutron grating interferometry (pnGI) setup used to perform the experiment (orange)

The spectra of the BOA beamline with and without the Be filter are shown in Fig. 4^44^. In addition the convolution of the Be-filtered spectrum with the wavelength-dependent visibility provided by the pnGI setup was calculated according to the method proposed by Harti et al.^48^ and it is shown in Fig. 4.

**Fig. 5:**
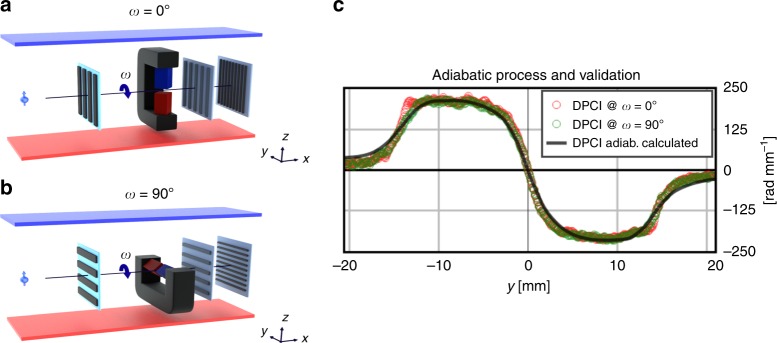
Adiabatic spin regime. Clarification of the adiabatic spin regime assumption for the two different configurations. **a** Sketch of the *ω* = 0° configuration with the grating lines and the homogeneous, well-defined, square-shaped and uniaxial magnetic field oriented along the initial neutron spin state and guide field. **b** Sketch of the *ω* = 90° configuration of the grating lines and the uniaxial magnetic field-oriented perpendicular to the initial neutron spin state and guide field. **c** Comparison and validation of the differential phase contrast image (DPCI) signals of the *ω* = 0°, *ω* = 90° configurations and the calculated one in the adiabatic spin regime assumption

Subsequently, the theoretical curve, based on the known magnetic field distribution, configuration *ω* = 0°, from the Hall probe measurement, Fig. 2b, and the measured line profile of the DPCI are in good agreement, Fig. 5c, validating the correct measurement of the magnetic field.

### Adiabatic spin regime in a horizontal-aligned magnetic field

The configuration *ω* = 0°, Fig. 5a, with the magnetic field vector aligned parallel to the polarization of the incoming beam, which was chosen for the initial experiment is, however, a simplification of the general case. The product of the vectors in Eq. (1) for the interaction potential equals the scalar product of the length of the vectors in this case. In a second approach, we tested a perpendicular alignment of the magnetic field vector to the initial polarization direction. In order to probe differential phase effects, we consequently also rotated the grating interferometer by 90°, corresponding to the configuration *ω* = 90° in Fig. 5b, being sensitive in the direction of the field gradient. According to Eq. (1) the potential would now become *V*_mag_(*r*) = 0 and hence also the effect of the magnetic field on the neutron phase would vanish. However, this is only true if the spin does not couple adiabatically with the continuous magnetic field transition from the guide field to the sample field. The results are presented together with the setup sketches in Fig. 5a–c and they clearly imply otherwise. The results, Fig. 5c, coincide with the initial measurement and calculations. This proves that the extend of the decay of the sample field is still reaching wide enough to fulfill the adiabatic condition for the neutron given neutron spectrum to enable the adiabatic turn of the spin vector resulting in an orientation parallel to the sample field. The limitation of this method due to the adiabatic spin coupling can be easily estimated by calculating the magnetic field gradient that fulfill Eq. (3). Given the following assumptions, which are consistent with the uniaxial vertically aligned magnetic field case: neutron wavelength of 4 Å and a magnetic field of 1 T. The resulting adiabatic parameter is reached for a field gradients of Δ*B*/Δ*x* ≈10^5^ T m^−1^, which are hard to find for magnetic stray field configurations. A realistic scenario where these strong field gradients can be achieved is, for example, within the domain walls of ferromagnets.

### Phase shift induced by an anisotropic magnetic field

**Fig. 6:**
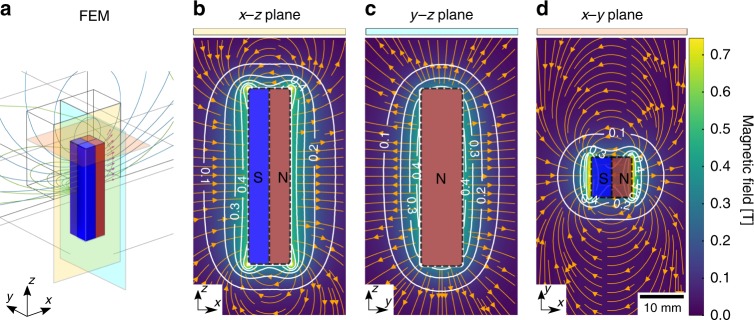
Inhomogeneous and anisotropic magnetic field simulations. **a** Rectangular cuboid neodymium permanent magnet characterization by three-dimensional (3D) finite element method (FEM) simulation of the magnetic field distribution, the three color-coded surfaces (yellow, light blue and red) represent the cross-section planes of the streamline plot. **b** *x*–*z* plane projection (yellow plane) of the magnetic field. **c** *y*–*z* plane projection (light blue plane) of the magnetic field. **d** *x*–*y* plane projection (red plane) of the magnetic field. The color code refers to the magnetic field vector’s magnitude

**Fig. 7:**
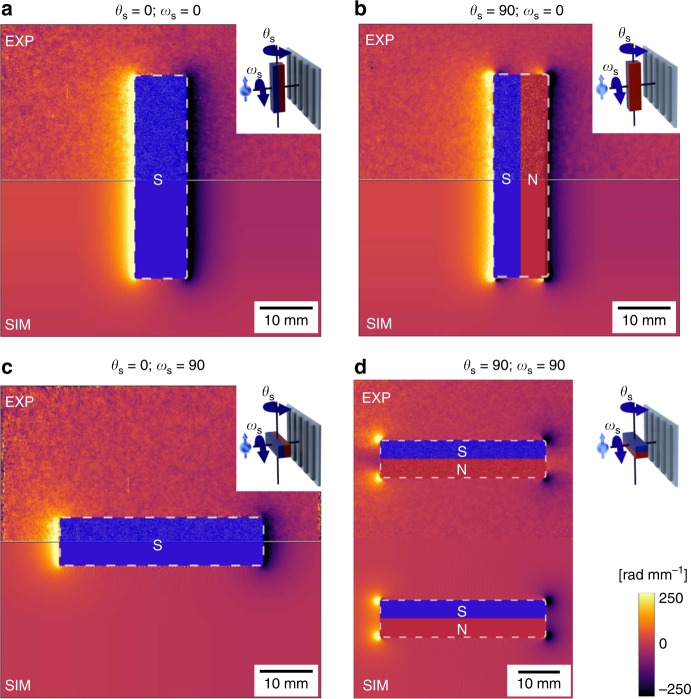
Experimental and calculated differential phase contrast image (DPCI) results of an inhomogeneous and anisotropic magnetic field. **a**–**d** Merged DPCI images of a rectangular cuboid neodymium permanent magnet. The insets in the upper right corners display the four different orientations of the permanent magnet according to the *θ*_s_ rotation rotation angle around the vertical axis as first, then the *ω*_s_ around the horizontal axis and keeping the gratings oriented parallel to the initial polarization according to the configuration *ω* = 0 of the homogeneous, well-defined, square-shaped, and uniaxial magnetic field. The four DPCI images are merged along the horizontal axis in order to aid the comparison between the experimental and the calculated data. The top halves have been retrieved from the experimental results, while the bottom ones have been calculated one from the finite element method (FEM) simulations. The dashed line represents the edges of the permanent magnet

**Fig. 8:**
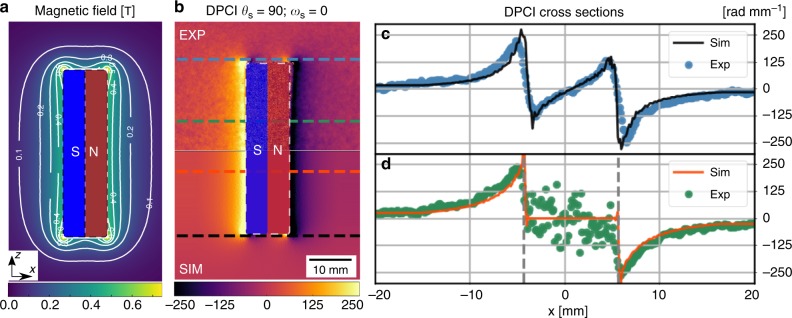
Quantitative analysis of an inhomogeneous and anisotropic magnetic field. Case: *θ*_s_ = 90°, *ω*_s_ = 0°. **a** Cross-section of the magnetic field distribution along the *x*–z plane the cross-section. **b** Differential phase contrast image (DPCI) images, the top one is the retrieved from the experimental data while the bottom one is calculated from the finite element method (FEM) simulations. The two horizontal line profiles are mirrored at the same heights in both experimental and simulated images. **c** Comparison plot of the horizontal line profile at the top/bottom faces highlighting the swirling of the magnetic field near the edge. **d** Comparison plot of the horizontal line profile at the middle height of the permanent magnet. The area within the vertical dashed lines shows the overlap with the neodymium permanent magnet where there is no transmission, and therefore no DPCI signal

In order to further generalize our approach from the previous well-defined, square-shaped, and uniaxial magnetic field as investigated, we subsequently considered a magnetic field having varying components and gradients in all the three orthogonal directions, **B** = (*B*_*x*_, *B*_*y*_, *B*_*z*_). The general case of an inhomogeneous and anisotropic magnetic field distribution, produced by a permanent magnet, assessed experimentally and modeled by finite element method (FEM) simulation, as depicted in Fig. 6. The pnGI results are summarized in Figs. 7 and 8. The considered and sampled magnetic field is produced by a rectangular cuboid, 40 × 10 × 10 mm^3^, of a neodymium permanent magnet (NdFeB). Figure 6a shows the magnetic field lines around the permanent magnet, which displays the typical dipole configuration and the corresponding direction of magnetization along the *x*-axis. The rectangular shape of the cuboid offers peculiar magnetic flux lines and a constant gradient characterizes its distribution along the long faces, as shown in Fig. 6b, c. A strong swirling emerges close to the short edges in Fig. 6b–d. Due to the magnetic field gradient distribution, the different neutron paths are characterized by changes in strength and direction of the magnetic field, covering all the scenarios of the general case.

The experimental results are shown in the top half of the images in Fig. 7a–d for the different sample configurations:

1. *θ*_s_ = 0°, *ω*_s_ = 0°, Fig. 7a,
2. *θ*_s_ = 90°, *ω*_s_ = 0°, Fig. 7b,
3. *θ*_s_ = 0°, *ω*_s_ = 90°, Fig. 7c,
4. *θ*_s_ = 90°, *ω*_s_ = 90°, Fig. 7d,

where *θ*_s_ is the rotation angle of the dipole around the vertical axis applied first and *ω*_s_ is the rotation around the horizontal axis, as depicted in the respective insets and with respect to the fixed incident polarization. In all four configurations the gratings have been kept oriented parallel to the initial spin state and to the guide field as shown in the insets of Fig. 7. This coincides with the initial measurement presented as *ω* = 0°, Fig. 5a, and a transverse sensitivity of the field inhomogeneity with respect to the grating lines. The constant magnetic field gradients measured along the long faces of the permanent magnet, shown in Fig. 6b, c, dominate the resulting images in Fig. 7a, b, while these are not visible in Fig. 7c, d due to their orientation parallel to the grating lines and the directional sensitivity of the pnGI setup in these cases. The effect on the DPCI signal of the swirling of the flux closure in the corners, visible in Fig. 6b, d, is depicted in Fig. 7b, d, while it does not emerge in Fig. 7c due to the permanent magnet *θ*_s_ = 0° and *ω*_s_ = 90° orientation in respect to the grating lines. The bottom halves of Fig. 7a–d show the calculated DPCI images from the FEM simulations. The DPCI images in Fig. 7a–c are merged along the horizontal symmetry axis. In Fig. 7d the whole permanent magnet is shown due to the different axes of symmetry of the data and the permanent magnet orientation. The DPCI images retrieved from the pnGI measurements show good agreement with the ones calculated from the FE M simulations for all the four orientations (Fig. 7). A more quantitative evaluation of the pnGI results for the case *θ*_s_ = 0° and *ω*_s_ = 90° is shown in Fig. 8. The magnetic field gradients distribution calculated, Fig. 8a, is reflected in the measured DPCI image in Fig. 8b. A direct comparison between the experimental and calculated signals is shown in Fig. 8c, d, where two line profiles at different heights in both experimental and simulated DPCI are plotted.

## Discussion

We have demonstrated and proven how pnGI yields quantitative DPCIs induced by magnetic fields. The experimental results obtained from the homogeneous, well-defined, square-shaped, and uniaxial magnetic field shows good agreement with calculations based on Hall probe measurements. This establishes a validation of the pnGI method for retrieving quantitative information about the phase effects of magnetic field interaction. The required adiabatic spin coupling to the sample field has been also experimentally demonstrated. The inhomogeneous and anisotropic magnetic field produced by a cuboid neodymium permanent magnet allowed us to extend the prove of the capability of the method to remotely probe strong magnetic fields with spatial resolution to the general case of magnetic fields of arbitrary inhomogeneous orientations and varying gradients. The experimental results are in all cases in good agreement with the calculated DPCI values. Further investigations are possible in many ways for the direct 3D reconstruction of the magnetic field distribution. This can be obtained either by combining the pnGI with a computed tomography approach or calculating the field distribution from the radiographic dataset and a priori knowledge of the sample geometry. The presented approach paves the way for investigations of magnetic fields characterized by strong fields and gradients previously inaccessible with the existing neutron imaging techniques and direct applications to a wide range of scientific and engineering challenges such as electric power systems and superconducting wires.

## Methods

### pnGI setup

The source grating *G*_0_ is a gadolinium aperture mask with transmitting slits with a periodicity of *p*_0_ = 1076 μm. *G*_0_ creates an array of periodically repeating coherent line sources, which fulfills the spatial coherence requirements for the interferometer formed by *G*_1_ and *G*_2_. The arrayed source thus decouples spatial resolution from spatial coherence. The silicon phase grating *G*_1_ with periodicity of *p*_1_ = 7.97 μm is placed at a distance of 5.23 μm downstream with respect to *G*_0_ and acts as a phase mask and imprints periodic phase interference pattern in the plane of the analyzer grating. The analyzer grating *G*_2_ with periodicity of *p*_2_ = 4 μm is made of Gadolinium and operated in the first Talbot order configuration with *d*_*t*1_ = 19.6 mm^45^. A cooled Be filter is installed upstream the interferometer to cut-off neutrons with a wavelength *λ* <4 Å; the spectrum is shown in Fig. 4. The neutron beam is polarized along the *z*-direction by a polarizing bender in the beam extraction system of the instrument^44,46^. All components are embedded into a guide field system in order to keep the polarization of the beam. The polarization of the beam with the described pnGI setup was measured to 90.5% corresponding to a flipping ratio of 20^2,44^. The images have been recorded using a 200-μm-thick ^6^Li/ZnS scintillator screen coupled to a digital camera [Andor iKon-M, 1024 × 1024 pixels] resulting in a spatial resolution of about 200 μm measured with a Siemens star test object^49^. A schematic of the pnGI setup is depicted in Fig. 1a.

### Hall probe measurements

The mapping of the magnetic field distribution in between the pole shoes has been performed with a 1D Hall probe by scanning its *z* component. We used a step size of 1.5 mm in both *x* and *y* directions and then applied a bilinear interpolation to the mesh data.

### FEM simulation

The FEM simulation of the magnetic field distribution, for the calculation of the induced phase shift for validation of the experimental results, has been performed with the software COMSOL Multiphysics with a mesh size of 0.25 mm.

## Supplementary information

Supplementary Information

Peer Review

## Data Availability

The data that support the findings of this study are available from the corresponding author upon reasonable request.

## References

[1] Greene GL (1979). Measurement of the neutron magnetic moment. Phys. Rev. D.

[2] Williams, W. G. *Polarized Neutrons* (Clarendon Press, Oxford, 1988).

[3] Lake Bella (2005). Quantum magnets show their hand. Nature Physics.

[4] Wiedenmann A (2005). Polarized SANS for probing magnetic nanostructures. Phys. B.

[5] Mühlbauer S (2009). Morphology of the superconducting vortex lattice in ultrapure niobium. Phys. Rev. Lett..

[6] Mühlbauer S (2019). Magnetic small-angle neutron scattering. Rev. Mod. Phys..

[7] Treimer W (2014). Radiography and tomography with polarized neutrons. J. Magn. Magn. Mater..

[8] Strobl M (2018). Topical review: Polarisation measurements in neutron imaging. J. Phys. D.

[9] Kardjilov N, Hilger A, Manke I, Strobl M, Banhart J (2018). Imaging with polarized neutrons. J. Imaging.

[10] Manke I (2010). Three-dimensional imaging of magnetic domains. Nat. Commun..

[11] Schulz M (2010). Towards a tomographic reconstruction of neutron depolarization data. J. Phys. Conf. Ser..

[12] Jorba P (2018). High-resolution neutron depolarization microscopy of the ferromagnetic transitions in Ni_3_Al and HgCr_2_Se_4_ under pressure. J. Magn. Magn. Mater..

[13] Kardjilov N (2008). Three-dimensional imaging of magnetic fields with polarized neutrons. Nat. Phys..

[14] Shinohara T (2011). Quantitative magnetic field imaging by polarized pulsed neutrons at J-PARC. Nucl. Instrum. Methods Phys. Res. Sect. A.

[15] Treimer W, Ebrahimi O, Karakas N, Prozorov R (2012). Polarized neutron imaging and three-dimensional calculation of magnetic flux trapping in bulk of superconductors. Phys. Rev. B.

[16] Treimer W, Ebrahimi O, Karakas N (2012). Observation of partial Meissner effect and flux pinning in superconducting lead containing non-superconducting parts. Appl. Phys. Lett..

[17] Treimer W, Ebrahimi O, Karakas N (2013). Imaging quantum mechanical effects in superconductors with polarized neutrons. Phys. Procedia.

[18] Tremsin AS (2015). Imaging of dynamic magnetic fields with spin-polarized neutron beams. N. J. Phys..

[19] Dhiman I (2017). Thermodynamics of Meissner effect and flux pinning behavior in the bulk of single-crystal LaSrCuO (*x* = 0.09). Phys. Rev. B.

[20] Piegsa F, van den Brandt B, Hautle P, Kohlbrecher J, Konter J (2009). Quantitative radiography of magnetic fields using neutron spin phase imaging. Phys. Rev. Lett..

[21] Strobl M (2009). Imaging with polarized neutrons. Phys. B.

[22] Sales M (2018). Three dimensional polarimetric neutron tomography of magnetic fields. Sci. Rep..

[23] Hilger A (2018). Tensorial neutron tomography of three-dimensional magnetic vector fields in bulk materials. Nat. Commun..

[24] Sales M (2019). Three dimensional polarimetric neutron tomography beyond the phase-wrapping limit. J. Phys. D.

[25] Strobl M, Treimer W, Walter P, Keil S, Manke I (2007). Magnetic field induced differential neutron phase contrast imaging. Appl. Phys. Lett..

[26] Pfeiffer F (2006). Neutron phase imaging and tomography. Phys. Rev. Lett..

[27] Strobl M, Harti R, Grünzweig C, Woracek R, Plomp J (2017). Small angle scattering in neutron imaging—a review. J. Imaging.

[28] Strobl M (2008). Neutron dark-field tomography. Phys. Rev. Lett..

[29] Grünzweig C (2008). Bulk magnetic domain structures visualized by neutron dark-field imaging. Appl. Phys. Lett..

[30] Grünzweig C (2008). Neutron decoherence imaging for visualizing bulk magnetic domain structures. Phys. Rev. Lett..

[31] Lee SW (2010). Observation of magnetic domains in insulation-coated electrical steels by neutron dark-field imaging. Appl. Phys. Express.

[32] Betz B (2016). Magnetization response of the bulk and supplementary magnetic domain structure in high-permeability steel laminations visualized in situ by neutron dark-field imaging. Phys. Rev. Appl..

[33] Betz B (2016). Frequency-induced bulk magnetic domain-wall freezing visualized by neutron dark-field imaging. Phys. Rev. Appl..

[34] Rauscher P (2016). The influence of laser scribing on magnetic domain formation in grain oriented electrical steel visualized by directional neutron dark-field imaging. Sci. Rep..

[35] Harti RP (2018). Dynamic volume magnetic domain wall imaging in grain oriented electrical steel at power frequencies with accumulative high-frame rate neutron dark-field imaging. Sci. Rep..

[36] Reimann T (2016). Neutron dark-field imaging of the domain distribution in the intermediate state of Lead. J. Low Temp. Phys..

[37] Reimann T (2015). Visualizing the morphology of vortex lattice domains in a bulk type-II superconductor. Nat. Commun..

[38] Reimann T (2017). Domain formation in the type-II/1 superconductor niobium: Interplay of pinning, geometry, and attractive vortex-vortex interaction. Phys. Rev. B.

[39] Strobl M (2014). General solution for quantitative dark-field contrast imaging with grating interferometers. Sci. Rep..

[40] Strobl M (2016). Wavelength-dispersive dark-field contrast: micrometre structure resolution in neutron imaging with gratings. J. Appl. Crystallogr..

[41] Harti, R. P. et al. Sub-pixel correlation length neutron imaging: spatially resolved scattering information of microstructures on a macroscopic scale. *Sci. Rep*. **7**, 44588 (2017). 10.1038/srep44588 PMC5355987 28303923

[42] Harti RP (2018). Visualizing the heterogeneous breakdown of a fractal microstructure during compaction by neutron dark-field imaging. Sci. Rep..

[43] Rauch, H. & Werner, S. A. *Neutron Interferometry* 2nd edn. (Oxford University Press, Oxford, 2015).

[44] Morgano M, Peetermans S, Lehmann E, Panzner T, Filges U (2014). Neutron imaging options at the BOA beamline at Paul Scherrer Institut. Nucl. Instrum. Methods Phys. Res. Sect. A.

[45] Grünzweig C (2008). Design, fabrication, and characterization of diffraction gratings for neutron phase contrast imaging. Rev. Sci. Instrum..

[46] Schulz M (2010). Comparison of polarizers for neutron radiography. J. Phys. Conf. Ser..

[47] Marathe S (2014). Improved algorithm for processing grating-based phase contrast interferometry image sets. Rev. Sci. Instrum..

[48] Harti RP (2017). Visibility simulation of realistic grating interferometers including grating geometries and energy spectra. Opt. Express.

[49] Grünzweig C, Frei G, Lehmann E, Kühne G, David C (2007). Highly absorbing gadolinium test device to characterize the performance of neutron imaging detector systems. Rev. Sci. Instrum..

