# Supplementary material for: Visualization and quantification of inhomogeneous and anisotropic magnetic fields by polarized neutron grating interferometry
Source: Nat Commun. 2019 Aug 22;10:3788. doi: 10.1038/s41467-019-11590-2 (PMC6706400; doi:10.1038/s41467-019-11590-2)
Supplement: Supplementary file 1 — Supplementary Information [file 41467_2019_11590_MOESM1_ESM.pdf]

Supplementary Information

Visualization and quantification of inhomogeneous and anisotropic magnetic fields by polarized neutron grating interferometry

J. Valsecchi[1,2], R. P. Harti[1,2], M. Raventós[1,2], M. D. Siegwart[1,3], M. Morgano[1], P. Boillat[1,3], M. Strobl[1,4], P. Hautle[5], L. Holitzner[5], U. Filges[5], W. Treimer[6], F. M. Piegsa[7], and C. Grünzweig*[1]

[1]Laboratory for Neutron Scattering and Imaging, Paul Scherrer Institut, Villigen, Switzerland
[2]University of Geneva, Geneva, Switzerland
[3]Electrochemistry Laboratory, Paul Scherrer Institut, Villigen, Switzerland
[4]University of Copenhagen, NBI, Copenhagen, Denmark
[5]Laboratory for for Scientific Developments and Novel Materials , Paul Scherrer Institut, Villigen, Switzerland
[6]University of Applied Sciences, Beuth Hochschule für Technik Berlin, Germany
[7]Laboratory for High Energy Physics, Albert Einstein Center for Fundamental Physics, University of Bern, Bern, Switzerland

*christian.gruenzweig@psi.ch

Supplementary Note 1: Phase-stepping method for pnGI data acquisition

Most of the Talbot-Lau interferometers, such as the presented pnGI, adopt the so-called phase-stepping approach. As indicated in Supplementary Figure 1(a) a stack of individual images with sample in place is recorded, referred as Sample Projection (smp), by scanning one of the gratings along the y_g direction, usually over an integer of a full period (y_1, $y_2 \ldots y_n$). An individual image is recorded at each discrete step of the grating stepping. Then the same stepping procedure is conducted without a sample in place, leading to a series of individual images that creates the Open Beam (OB) stack. The intensity modulation leads to sinusoidal oscillations in each pixel of the Sample Projections (indicated by green dots) and the OBs (indicated by red dots), as shown in Supplementary Figure 1(b). The pixel-wise comparison of the OB modulation (red) witeh the Sample Projection modulation (green) for each individual pixel (i, j) leads to the extraction of the TI and DPCI, Supplementary Figure 1(b,c). In order to do so the intensities of a single pixel in an individual image recorded at the indicated grating step y_g are fitted with a sinusoidal function with the parameters mean value $D(i, j)$ and the interferometric phase $\theta(i, j)$. The TI signal for an individual pixel TI(i, j) is produced by evaluating the mean value D after a stepping procedure with sample $D_{smp}(i, j)$ and a stepping procedure without the sample in the beam $D_{OB}(i, j)$. Thus the transmission value for the individual (i, j)-pixel is defined by:

$$TI\big(i,j\big) = \frac{D_{smp}(i,j)}{D_{OB}(i,j)} \tag{1}$$

The DPCI(i, j) is also extracted by direct comparison of the interferometric phase of the stepping with sample $\theta_{smp}(i, j)$ and the phase of the open beam stepping $\theta_{OB}(i, j)$, while taking phase wrapping into account. DPCI(i, j) is determined by:

$$DPCI\big(i,j\big) = \theta_{smp}(i,j) - \theta_{OB}(i,j) = \Delta\theta(i,j) \tag{2}$$

as shown in Supplementary Figure 1(b).

It is important to keep in mind that the retrieved interferometric phase is the resulting sum of the phases induced by all the different potentials, such as the nuclear and the magnetic. In order to discriminate between the contributions to the phase shift induced by the nuclear interaction, which is not spin dependent, and the magnetic one,which is spin dependent both neutron spin states has to be measured. In this case the sample interferometric phase can be expressed in terms of the two possible neutron spin states up $|\uparrow\rangle$ and down $|\downarrow\rangle$:

$$\begin{aligned}\theta_{smp}^{|\uparrow\rangle}(i,j) &= \theta_{smp}^{nucl}(i,j) + \theta_{smp}^{mag}(i,j)\\ \theta_{smp}^{|\downarrow\rangle}(i,j) &= \theta_{smp}^{nucl}(i,j) - \theta_{smp}^{mag}(i,j)\end{aligned} \tag{3}$$

The evaluation of DPCI$_{mag}$ due to magnetic interaction is then retrieved by the average of the difference between the two spin states measurements, defined by:

$$DPCI_{mag}\big(i,j\big) = \frac{\Delta\theta^{|\uparrow\rangle}(i,j) - \Delta\theta^{|\downarrow\rangle}(i,j)}{2} \tag{4}$$

where $\Delta\theta^{|\uparrow\rangle}(i,j)$ and $\Delta\theta^{|\downarrow\rangle}(i,j)$ refer to the combination of Supplementary Equation (3) into Supplementary Equation (2). Once the DPCI$_{mag}$ expressed in unit of interferometric phase θ has been calculated, the resulting DPCI$_{mag}$ in terms of the neutron wave packet's phase shift Φ can be retrieved according to the following equation:

$$\theta = \frac{\lambda d_t}{p}\frac{\partial\Phi}{\partial y} \tag{5}$$

where λ is the wavelength, d_t the Talbot distance and p the stepped grating period.

The PCI(i, j) is then retrieved by transversally integrating the DPCI(i, j) according to the directional sensitivity of the pnGI setup.

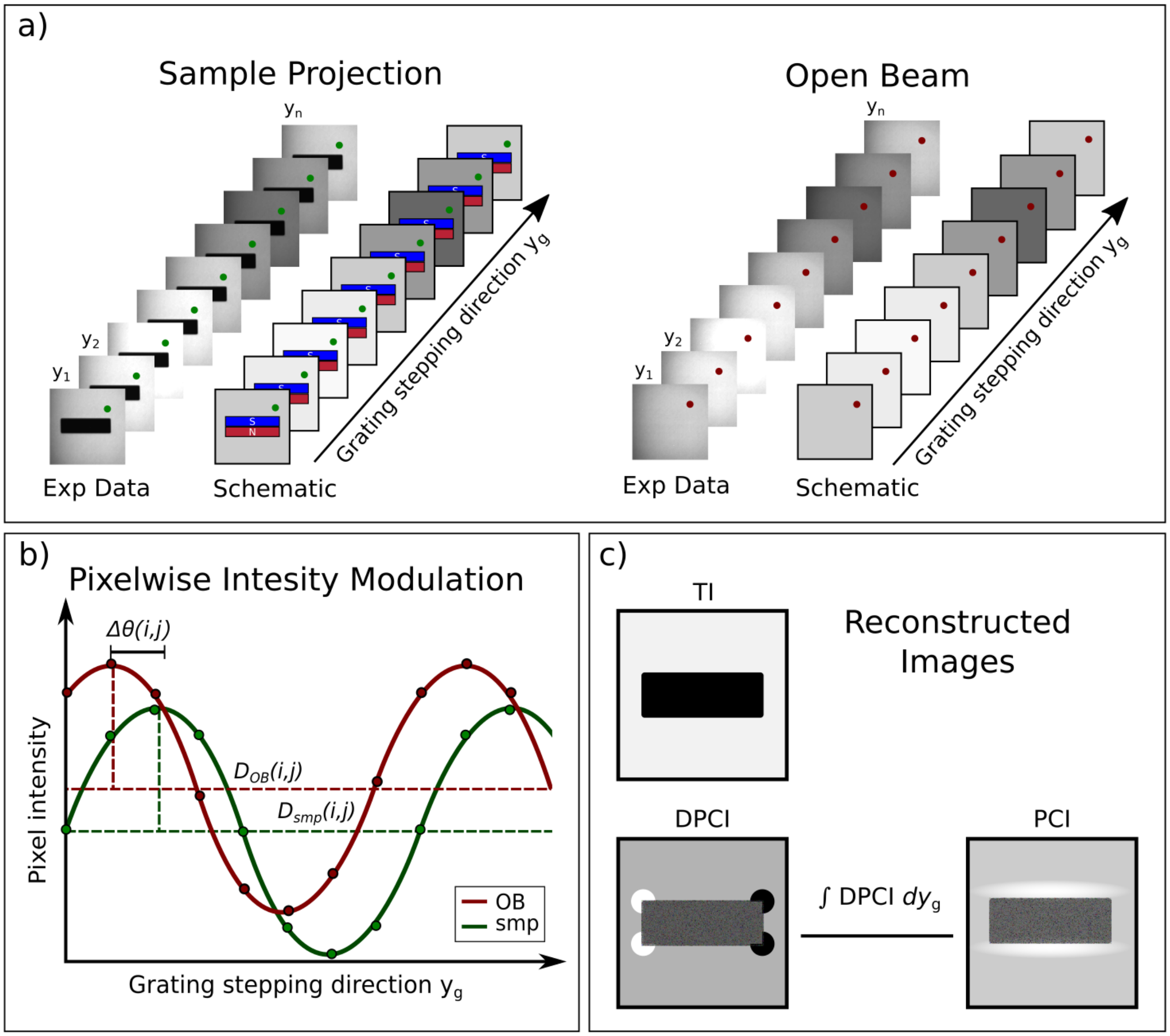

Supplementary Fig. 1 | Phase-stepping method for pnGI data acquisition. Schematic illustration of the data generation and processing procedure in neutron grating interferometry. a) A set of n-individual images with sample as well as without sample at various grating steps y_g are recorded. Green indicates the stack of recorded images for the Sample Projection and red the Open Beam respectively. The double stacks of images, for both sets, depict a real experimental dataset (Exp Data), as a practical example, and a simplified ones to lay out the nGI phase-stepping approach. The dot marker depicts the (i, j)-pixel on the matrix detector. b) The resulting intensity modulation for the (i, j)-pixel, green and red dots for the Sample Projection and the Open Beam respectively, as a function of the grating steps y_g and the corresponding a sinusoidal fits. $D_{smp}(i, j)$ and $D_{OB}(i, j)$ are the mean values obtained after the stepping procedure with and without the sample, respectively. $\Delta\theta(i, j)$ is the difference between the phase of the open beam stepping and the phase of the stepping with sample, while taking phase wrapping into account. c) The modulations in each pixel can be analyzed to result in a set of images. The transmission image (TI), the differential phase contrast image (DPCI) and the corresponding integrated phase contrast image (PCI).
