# Supplementary material for: Visualization and quantification of inhomogeneous and anisotropic magnetic fields by polarized neutron grating interferometry
Source: Nat Commun. 2019 Aug 22;10:3788. doi: 10.1038/s41467-019-11590-2 (PMC6706400; doi:10.1038/s41467-019-11590-2)
Supplement: Supplementary file 2 — Peer Review [file 41467_2019_11590_MOESM2_ESM.pdf]

Reviewers' comments:

Reviewer #1 (Remarks to the Author):

This is an outstanding work by a talented team. This manuscript could become a milestone publication in the area of magnetic field imaging, an area which has fundamental importance to electric power systems.

However, the presentation could be improved. Let’s start with the word “phase”. In this work, it has at least three distinct meanings: (1) The phase of a sinusoidal signal measured at a pixel as a function of grating step motion. (2) The wavefront structure created at the first Talbot distance. (3) The neutron precessional motion. The reviewer objects to the intertwined usage of phase, especially in equation 4 and the preceding sentence, unless a better introduction is given.

A suggestion for an introduction is addition of a discussion which replaces G0 with a single, narrow horizontal slit. I would like to see the authors develop TI Fig 3c, DPCI Fig 3e, and PCI Fig 3g for the single-slit, spin-up polarized neutron beam through the square profile, 45 degree orientation magnetic field in the adiabatic regime. This reviewer admits to some uncertainty in the TI and DPCI predictions, even after a review of the Stern-Gerlach experiment and consideration of the negative sign of the neutron magnetic moment.

Minor issues:
Fig 1 axes triad probably has xy incorrect relative to Fig 3
Fig 3d,e show no phase wrap (in the sinusoidal solution sense). How can this be?
In the paragraph that starts with “A common feature of imaging techniques…”, please change"
"This technique relays on the ..” to “relies”
“…imaging method its mayor.. “ to “major”

Reviewer #2 (Remarks to the Author):

This paper introduces a neutron phase-contrast method designed to image high magnetic field gradients. The authors demonstrate their method by imaging the magnetic field induced by a permanent magnet, and by comparing the resulting data to model calculations. I see no reason to doubt the technical validity of the experiments and the statements by the authors. However, I do not believe that the paper is suitable for publication in Nature Communications, because the methodological improvements appear incremental in contrast to prior work, and because the results lack general interest. In particular, the authors have only demonstrated imaging of a very simple field configuration, and they have not made the case that their methodological improvements will enable important new experiments that are not feasible otherwise. The paper is clearly of interest mostly to specialists and should be published in a more specialized journal.

Reviewer #3 (Remarks to the Author):

The authors report on the visualization and characterization of magnetic fields by polarized neutron grating interferometry in the adiabatic spin regime.
The working principle of this method is demonstrated by measuring the differential phase contrast images of an uniaxial vertical-aligned magnetic field and an inhomogeneous and anisotropic magnetic field created by permanent magnets.
It is an interesting paper introducing a novel neutron grating interferometry using polarized neutrons and would warrant publication in Nature Commun., after the authors have considered following points.

1) Since not all the readers of Nature Commun. are experts on interferometry, a brief description of the actual measurement procedures, such as ’phase-stepping approach’ (on line 8 from the bottom of p. 3) would be at place. A short description in the main text with the more detailed explanation of the data reduction to obtain DPCI and PCI in a supplemental material can be a solution.

2) Although the paper reports on a demonstration of principle and hence presents only the excellent agreement between the experimental and finite elements calculation results, the possible applications including directive outlook on the reconstruction of the field distribution using this method.

guideline for field distribution reconstruction and possible application cases should be indicated in the final paragraph.

3) The limitation of this method for application due to the required adiabatic spin coupling should be stated more quantitatively. Especially the field distributions in magnetic materials are often associated with the non-adiabatic conditions and hence would probably limit the application.

4) In Fig. 6 b) through d) the arrows of the vectors are difficult to recognize.

5) For the presentation of the figures 7 it would be more illuminating if the experimental and calculated results can be explained using the field distributions shown in the figures 6 considering the directional sensitivity of the pnGI setup.

6) In Fig. 7 d) the comparison of experimental and calculated results in upper and lower half facing S and N, respectively seems to indicate, that the DPCI results does not change by exchanging S and N in this case. Does this mean that in Theta_s = 90 case, omega = 90 and -90 gives the same DPCI distribution?

7) On line 7 from the bottom of p. 3 it is stated ‘both spin states are reconstructed with …’. But the results presented are only for one spin state and the need for measuring both spin states is not obvious.

Typos:

Reference 45 should read
‘…. Rev. Sci. Instrum. 79, 053703, DOI: …..’

Reply to Referee #1

We want to thank the Referee for her/his report. We are pleased to hear that the Referee finds our work "*outstanding*", and that "*this manuscript could become a milestone publication in the area of magnetic field imaging*" and hence supports a publication in Nature Communications.

In the following, we provide a point to point response to her/his comments:

Comment #1:

"This is an outstanding work by a talented team. This manuscript could become a milestone publication in the area of magnetic field imaging, an area which has fundamental importance to electric power systems. However, the presentation could be improved. Let's start with the word "phase". In this work, it has at least three distinct meanings: (1) The phase of a sinusoidal signal measured at a pixel as a function of grating step motion. (2) The wavefront structure created at the first Talbot distance. (3) The neutron precessional motion. The reviewer objects to the intertwined usage of phase, especially in equation 4 and the preceding sentence, unless a better introduction is given."

Reply to comment #1:

We thank the Referee for this constructive feedback and we therefore revised our manuscript to overcome this intertwined usage of the word "phase". In our revised manuscript we added two subfigures (b) and (c) in Fig. 1.
Subfigures (b) is used to show the phase shift of the neutron wavefront induced by the interaction with a phase object which leads to a refraction angle.
Subfigures (c) depicts the perturbations of the incident neutron wavefront, induced by refraction on a phase object in the beam, which lead to local displacement of the fringes in a Talbot-Lau interferometer.
We attempted to use a more precise language, rephrasing it in order to clarify whether or not it's an interferometric phase (1), , or neutron wave packet's phase shift (2), .
Additionally, we provide a Supplementary Information document which cover the phase-stepping procedure and depicts the terminology of the interferometric phase and its connection to the neutron wave packet's phase shift.
The term "phase" in relation to the Larmor's precession meaning (3) has been used only a reference to the nomenclature used in the reference [20].
We are convinced that our changes to the manuscript solved the intertwined usage of the word "phase".

Comment #2:

"A suggestion for an introduction is addition of a discussion which replaces G0 with a single, narrow horizontal slit. I would like to see the authors develop TI Fig 3c, DPCI Fig 3e, and PCI Fig 3g for the single-slit, spin-up polarized neutron beam through the square profile, 45 degree orientation magnetic field in the adiabatic regime. This Reviewer admits to some uncertainty in the TI and DPCI predictions, even after a review of the Stern-Gerlach experiment and consideration of the negative sign of the neutron magnetic moment."

Reply to comment #2:

The spatial coherence of the beam is provided by G0 which only creates an array of individually coherent sources, its period is matched with the other nGI setup parameters and its lines are parallel to G1 and G2 ones. In the case of Fig. 3 G0 can be replaced with a vertical slit without affecting the functional principle of the interferometer and TI, DPCI, and PCI results. The Referee suggests to replace G0 with a narrow horizontal slit. In this case the spatial coherence requirements will not be fulfilled by the horizontal slit and the Talbot-Lau interferometer won't work. Hence, it won't be possible to retrieve DPCI and consequently PCI, while it will still be possible to measure TI. Moreover, the Referee suggests to consider the Stern-Gerlach experiment in analogy with the one presented in our manuscript. The Stern-Gerlach experiment reveals the space quantization between two spin states induced by an inhomogeneous magnetic field of a particle carrying angular momentum. The observed deflection is proportional to the magnetic field gradient. In the case presented in Fig. 3 of our manuscript it has to be considered that the probed sample is characterized by a homogeneous and uniaxial magnetic field and the neutron beam is polarized therefore a spatial splitting cannot be observed due to the two quantum states.

Minor issues #1:

"Fig 1 axes triad probably has xy incorrect relative to Fig 3 "

Reply to minor issues #1:

We thank the Referee for pointing out this incorrect labelling and we corrected the labelling of Fig.3(c,e,g).

Minor issues #2:

“Fig 3(d,e) show no phase wrap (in the sinusoidal solution sense). How can this be?”.

Reply to minor issues #2:

The DPCI results of Fig.3(d,e) are expressed in $\Delta\Phi/\Delta y$ [rad/mm] units, retrieved from the original unwrapped DPCI in interferometric phase θ units, according to Eq.4. The same data processing applies to Fig.7 and Fig.8.

Typos:

"This technique relays on the ...” to “relies”

“…imaging method its mayor...” to “major”

We thank the Referee for the corrections.

Reply to Referee #2

We wish to thank Referee #2 for her/his efforts to review our manuscript. We are pleased to hear that the Referee has no doubt about "*the technical validity of the experiments and the statements by the authors*".
The Referee finds several points of criticism and hence does not support a publication of our manuscript.

In the following, we provide a point to point response to her/his comments:

Comment #1:

"This paper introduces a neutron phase-contrast method designed to image high magnetic field gradients. The authors demonstrate their method by imaging the magnetic field induced by a permanent magnet, and by comparing the resulting data to model calculations. I see no reason to doubt the technical validity of the experiments and the statements by the authors. However, I do not believe that the paper is suitable for publication in Nature Communications, because the methodological improvements appear incremental in contrast to prior work, and because the results lack general interest. In particular, the authors have only demonstrated imaging of a very simple field configuration, and they have not made the case that their methodological improvements will enable important new experiments that are not feasible otherwise. The paper is clearly of interest mostly to specialists and should be published in a more specialized journal. "

Reply to comment #1, regarding "the methodological improvements appear incremental in contrast to prior work":

In the following we provide a list with high-impact references for both the neutron grating interferometry technique and polarized neutron imaging showing how typical methodological improvements are reported. We consider the methodological improvements presented in our manuscript at least the same impact. Especially, we want to point out to the referee, that in contrast to all previous nGI works and the high impact publications listed below where the experiments have been performed by means of unpolarized nGI setups, our manuscript report on the first polarized nGI setup that allows to visualize magnetic phase shift.

List of high impact publications related to **unpolarized neutron grating interferometry** in the last years:

- *[26] Pfeiffer, F. et al. Neutron phase imaging and tomography. Phys. Rev. Lett. 96, 215505, DOI: 10.1103/PhysRevLett.96. 215505 (2006).*

Milestone publication introducing the first unpolarized 2D and 3D nGI experiment visualizing the nuclear phase shift.

- *[28] Strobl, M. et al. Neutron dark-field tomography. Phys. Rev. Lett. 101, DOI: 10.1103/PhysRevLett.101.123902 (2008).*

  First unpolarized nGI dark-field tomography.

- *[30]. Grünzweig, C. et al. Neutron decoherence imaging for visualizing bulk magnetic domain structures. Phys. Rev. Lett. 101, DOI: 10.1103/PhysRevLett.101.025504 (2008).*

  First unpolarized nGI dark-field radiography on magnetic domain systems.

- *[10] Manke, I. et al. Three-dimensional imaging of magnetic domains. Nat. Commun. 1, 125, DOI: 10.1038/ncomms1125 (2010).*

  First unpolarized nGI dark-field tomography on magnetic domain systems.

- *[37] Reimann, T. et al. Visualizing the morphology of vortex lattice domains in a bulk type-II superconductor. Nat. Commun. 6, 8813, DOI: 10.1038/ncomms9813 (2015). 1308.3612.*

  First unpolarized nGI dark-field radiography on type-II superconductors.

List of high impact publications related to **polarized neutron imaging** in the last years:

- *[13] Kardjilov, N. et al. Three-dimensional imaging of magnetic fields with polarized neutrons. Nat. Phys. 4, 399-403, DOI:10.1038/nphys912 (2008).*

  New 2D and 3D polarized neutron imaging on magnetic and superconducting systems.

- *[22] Sales, M. et al. Three Dimensional Polarimetric Neutron Tomography of Magnetic Fields. Sci. Rep. 8, 2214, DOI:10.1038/s41598-018-20461-7 (2018). 1704.04887.*

First 3D polarimetric time-of-flight neutron imaging on magnetic systems.

- *[23] Hilger, A. et al. Tensorial neutron tomography of three-dimensional magnetic vector fields in bulk materials. Nat. Commun. 9, 4023, DOI: 10.1038/s41467-018-06593-4 (2018).*

  3D polarimetric neutron imaging on magnetic and superconducting systems.

We also invite the Reviewer to consider the opinions of the other Reviewer:

Referee #1 pointing out that our manuscript is **"… a milestone publication in the area of magnetic field imaging…**".

Referee #3 states that our work is "**…an interesting paper introducing a novel neutron grating interferometry using polarized neutrons…**".

<u>Reply to comment #1, regarding "the results lack general interest":</u>

We disagree on this opinion, as the list of high impact references above especially for imaging magnetic phenomena shows that general interest if given.

Additionally, we want to cite one of the other referees. Referee #1 clearly asserts that our manuscript is "**… is an outstanding work … a milestone publication in the area of magnetic field imaging, … has fundamental importance …**".

<u>Reply to comment #1, regarding "the authors have only demonstrated imaging of a very simple field configuration, and they have not made the case that their methodological improvements will enable important new experiments that are not feasible otherwise.":</u>

We want to point out to the referee, that we first presented the results of a simple homogeneous, well defined, square shaped and uniaxial magnetic field distribution produced by a magnetic yoke. However, we than further extend our investigations to a much more complex magnetic field distribution with a inhomogeneous and anisotropic field configuration as produced by permanent magnets.
We also provide an extensive explanation of the adiabatic coupling of spin rotation, which let the reader grasp the whole picture of the undergoing physical phenomena and it allows to identify the application boundary of the pnGI approach.

We encourage the reviewer to recognize that the presented magnetic fields are characterized by strong fields and gradients previously inaccessible with the existing polarized neutron imaging techniques.

Reply letter to Referee #3

We want to thank Referee #3 for her/his efforts to review our manuscript. We are pleased to hear that the Referee states that our work "*would warrant publication in Nature Commun.*".
In the following, we provide a point to point response to her/his comments:

Comment #1:

"Since not all the readers of Nature Commun. are experts on interferometry, a brief description of the actual measurement procedures, such as 'phase-stepping approach' (on line 8 from the bottom of p. 3) would be at place. A short description in the main text with the more detailed explanation of the data reduction to obtain DPCI and PCI in a supplemental material can be a solution."

Reply to comment #1:

According to Referee's suggestions a brief description in the main text followed by an detailed Supplementary Information document have been added to describe in detail the data acquisition using the phase-stepping approach and the final data processing.

Comment #2:

"Although the paper reports on a demonstration of principle and hence presents only the excellent agreement between the experimental and finite elements calculation results, the possible applications including directive outlook on the reconstruction of the field distribution using this method. Guideline for field distribution reconstruction and possible application cases should be indicated in the final paragraph."

Reply to comment #2:

We thank the Referee for this constructive feedback. We added the following sentences to the conclusion and outlook section:

- Further investigations are possible in many ways for the direct 3D reconstruction of the magnetic field distribution. This can be obtained either by combining the pnGI with a computed tomography approach or calculating the field distribution from the radiographic dataset and a priori knowledge of the sample geometry.

- The presented approach paves the way for investigations of magnetic fields characterized by strong fields and gradients previously inaccessible with the existing neutron imaging techniques and direct applications to a wide range of scientific and engineering challenges such as electric power systems and superconducting wires.

Comment #3:

"The limitation of this method for application due to the required adiabatic spin coupling should be stated more quantitatively. Especially the field distributions in magnetic materials are often associated with the non-adiabatic conditions and hence would probably limit the application."

Reply to comment #3:

We thank the Referee for this constructive feedback. The required adiabatic spin coupling condition is not fulfill for field gradients stronger than $\Delta B/\Delta x \approx 10^5$ T/m, as calculated according to Eq.3 in the manuscript for a wavelength of 4 Å and a magnetic field of 1 T. Such strong gradients are typically hard to achieve for magnetic stray field distribution. In most cases the adiabatic spin coupling condition is fulfilled. An exceptional realistic scenario where such strong gradients can be found are inside ferromagnetic materials (passage from one domain to the other). These considerations have been added to the main text.

Comment #4:

"In Fig. 6 b) through d) the arrows of the vectors are difficult to recognize."

Reply to comment #4:

According to Referee's suggestions, Fig. 6 has been changed for a better visualization of the magnetic field distribution into a "streamline plots".

Comment #5:

"For the presentation of the figures 7 it would be more illuminating if the experimental and calculated results can be explained using the field distributions shown in the figures 6 considering the directional sensitivity of the pnGI setup."

Reply to comment #5:

We thank the Referee for this constructive feedback. We revised the main text and improved the readability for a better explanation of the results presented in Fig.7 by including the information about the field distribution of Fig.6 together the directional sensitivity of the pnGI setup.

Comment #6:

"In Fig. 7 d) the comparison of experimental and calculated results in upper and lower half facing S and N, respectively seems to indicate, that the DPCI results does not change by exchanging S and N in this case. Does this mean that in θ_s = 90 case, ω_s = 90 and -90 gives the same DPCI distribution?"

Reply to comment #6:

We thank the Referee for this very constructive hint. Accordingly, Fig. 7 (d) has been revised by depicting the different axes of symmetry of the data and the permanent magnet orientation. The results will be then reverse in case of θ_s = 90, ω_s = -90, or by keeping the θ_s = 90, ω_s = 90 orientation and flipping the beam polarization.

Comment #7:

"On line 7 from the bottom of p. 3 it is stated 'both spin states are reconstructed with …'. But the results presented are only for one spin state and the need for measuring both spin states is not *obvious."*

Reply to comment #7:

In our manuscript the presented results are characterized by phase shifts which are only induced by the magnetic potential. Flipping the polarization leads to the reversed results, e.g. the DPCI in Fig.3(d) will be inverted, therefore only one spin state is shown. Nevertheless, acquiring both spin states is crucial for a pnGI measurement in order to discriminate between the phase shift induced by the nuclear interaction, which is not spin dependent, and the magnetic one, which is spin dependent and to characterize the polarization of the neutron beam. A detailed formulation of the methodological approach is discussed in the Supplementary Information document and mentioned in the main text.

Minor issues #1:

Reference 45 should read '…. Rev. Sci. Instrum. 79, 053703, DOI: …..'

Reply to minor issues #1:

We thank the Referee for the correction.

REVIEWERS' COMMENTS:

Reviewer #1 (Remarks to the Author):

The major claim of this work is that polarized neutron grating interferometry yields quantitative differential phase contrast images that can be integrated to map the magnetic field. The comparison between experiment and theory uses two magnetic field systems, both studied in multiple orientations. The agreement between simulations and experiment is excellent. The mapping does require low velocity neutrons (cold) and low magnetic field gradients; both conditions are met in this experiment and for most envisioned applications, excepting the imaging of internal magnetic domains in ferromagnets as specifically pointed out by the authors.

In summary, the work is detailed, well described, and broadly applicable. This work is also at the same high quality and impact as recent neutron grating interferometry works published in high impact journals such as Nature Communications. Therefore, publication of this work, in the present form, is highly recommend in this journal.

Reviewer #2 (Remarks to the Author):

The authors have answered the technical queries competently. However, I remain unconvinced that this manuscript satisfies the general-interest criterion for publication in Nature Communications. In introducing a new scientific method in a high-impact journal, one typically shows that the method can solve a problem that could not previously been solved. The authors argue that the neutron-imaging field has a different tradition and cite several articles as evidence. While it is true that these papers don’t report the solution of a frontier research problem, some get considerably closer than the present paper, which just reports mapping of fields generated by permanent magnets. For instance, Ref. 23 reports the magnetic flux distribution in a superconductor that was not a-priori known. The authors of the present paper mention experiments on superconducting wires as a possible application of their method, but do not demonstrate its usefulness in this domain of investigation.

Having said this, I recognize that there is no hard criterion for general interest, and I do not wish to stand in the way of publication if the editors and the other referees that this criterion is satisfied.

Reviewer #3 (Remarks to the Author):

The authors have provided a comprehensive point-by-point response to referees’ reports and revised the manuscript accordingly including the revisions of figures. The addition of the detailed Supplementary Information on the data acquisition using the phase-stepping approach and the necessity of the data acquisition in both spin states nicely illustrates the essence of the method to broader communities of interested readers.
The revised manuscript thus definitely warrants publication in Nature Communications.
